# Antidiabetic activity, glucose uptake stimulation and α-glucosidase inhibitory effect of *Chrysophyllum cainito* L. stem bark extract

**DOI:** 10.1186/s12906-018-2328-0

**Published:** 2018-10-01

**Authors:** Hau Van Doan, Siriporn Riyajan, Roongtip Iyara, Nuannoi Chudapongse

**Affiliations:** School of Preclinical Sciences, Institute of Science, Suranaree University of Technology, Nakhon Ratchasima, 30000 Thailand

**Keywords:** *Chrysophyllum cainito*, Antidiabetic activity, Glucose uptake, α-glucosidase, Alloxan

## Abstract

**Background:**

*Chrysophyllum cainito* L., a tropical fruit tree, has been used as an alternative medicine for the treatment of diabetic patients in many countries. However, there is very limited scientific rationale for this medical use. The present study aimed to evaluate the antidiabetic activity of the extract from *C. cainito* stem bark and the possible mechanisms underlying this activity.

**Methods:**

Phytochemistry and in vitro antioxidant capacity of the extract were studied. Hypoglycemic activity of the extract was examined in normal and alloxan-induced diabetic mice. The effect of *C. cainito* extract on glucose absorption and glucose uptake were conducted using mouse isolated jejunum and abdominal muscle, respectively. Finally, an in vitro effect of *C. cainito* extract on α-glucosidase activity was evaluated.

**Results:**

*C. cainito* extract possessed a strong antioxidant activity comparable to the ascorbic acid and butylated hydroxytoluene. The extract at 500 mg/kg significantly reduced the area under curve of blood glucose level in oral glucose tolerance test in normal mice. In alloxan-induced diabetic model, similar to glibenclamide, a single dose of the extract significantly decreased fasting blood glucose level from 387.17 ± 29.84 mg/dl to 125.67 ± 62.09 mg/dl after 6 h of administration. From the isolated jejunum experiment, the extract at any doses used did not inhibit glucose absorption. However, the extract at 50 μg/ml significantly increased the amount of glucose uptake by abdominal muscles in the presence of insulin (*P* < 0.05). Lastly, it was found that the extract produced stronger inhibition of α-glucosidase activity (IC_50_ = 1.20 ± 0.09 μg/ml) than acarbose (IC_50_ = 198.17 ± 4.74 μg/ml).

**Conclusion:**

Direct evidence of antidiabetic activity of *C. cainito* stem bark with possible modes of action, glucose uptake stimulation and α-glucosidase inhibitory effect, was reported for the first time herein. These data support the potential use of this plant for the treatment of diabetic patients.

## Background

Diabetes mellitus, one of the most common metabolic disorders, has been reported to affect approximately 415 million people worldwide in 2015 and the number of cases has been estimated to increase to 642 millions in 2040 [1]. Chronic hyperglycemic patients have been living with a high risk of macrovascular complications (e.g., coronary artery disease, peripheral arterial disease, and/or stroke) and microvascular complications (e.g., retinopathy, nephropathy, and neuropathy) [2]. Being supplied with high blood glucose, cells can generate the formation of free radicals and reactive oxygen species. In turn, an overload of free radicals can damage cellular macromolecules including lipid, protein and nucleic acids leading to the progression of diabetes and the development of its complications. Therefore, the antioxidant therapy is one of the important therapeutic strategy in diabetes management [3]. Plants have been widely accepted that they provide natural antioxidant compounds [4, 5]. In addition, plant products and their derivatives also possess many other pharmacological activities, such as anti-inflammatory, antimicrobial, anticancer and antidiabetic activity. Thus, traditional medicines have been proved to be a vital source of future drugs to counteract many diseases including diabetes mellitus [6].

*Chrysophyllum cainito* L. (commonly known as Star Apple) is a tropical fruit tree of which many biological activities have been demonstrated. The documented benefits of *C. cainito* include antihypertensive, anti-inflammatory [7], antioxidant and wound healing [8], antibacterial [9] and antidiabetic activity [10, 11]. The stem bark decoction has been traditionally used as tonic, stimulant, antidiarrheals [12] and antidiabetics [13]. Although several parts of *C. cainito*, such as fruit, leaf and stem, have been used as alternative medicines for the treatment of diabetic patients in many countries, there is limited pharmacological basis for this therapeutic application. The present study was carried out to evaluate the antidiabetic activity of the aqueous extract of *C. cainito* stem bark in animal models. In addition, the effects on glucose absorption, glucose uptake and α-glucosidase activity were also examined as the possible mechanisms underlying antidiabetic property of the extract.

## Methods

### Chemicals

2,2-Diphenyl-1-picrylhydrazyl (DPPH), 2,2′-Azino-bis (3-ethylbenzothiazoline-6-sulfonic acid) diammonium salt (ABTS), 2,4,6-tripyridyl-s-triazine (TPTZ), Peroxidase-glucose oxidase (PGO) enzyme, α-glucosidase, alloxan monohydrate were purchased from Sigma-Aldrich (MO, USA). Folin–Ciocalteu reagent was purchased from Carlo-Erba (Val de Reuil Cedex, France). Blood glucose test strips were purchased from Terumo (Tokyo, Japan).

### Plant extraction

The stem bark of *C. cainito* was collected from Mo Cay Nam district, Ben Tre, Vietnam. Plant verification was performed by Dr. Santi Watthana, a plant taxonomist, School of Biology, Institute of Science, Suranaree University of Technology, Thailand. Voucher specimens of leaf, fruit, flowers, and stem was stored at Suranaree University of Technology Botanical Garden under collected number H.DOAN-1. The bark was dried under the shade for a week before ground. In this study, water was chosen as an extraction solvent because water formulations are safe for human consumption compared to other organic solvents. It also increases bioavailability of active compounds. Furthermore, water maceration and decoction of this plant have been used by Vietnamese for treatment of diabetic patients. We have chosen simple maceration rather than decoction to prevent chemical degradation from high temperature. Briefly, 50 g of ground material were shaken with 200 ml of deionized water at room temperature for 2 h. The process was repeated four times. The combined extract was centrifuged at 5000 rpm for 15 min to remove solid residue. The supernatant was evaporated and dried by lyophilizer. The extract of *C. cainito* stem bark (CE) was kept at -20 °C until used for the experiments.

### Phytochemical screening and total phenolic content determination

Phytochemical screening for tannin, phenols, alkaloids, flavonoids, saponin, steroids and glycosides were conducted as previously described [14]. The presence of terpenoids was also examined [15].

Total phenolic content was determined by Folin–Ciocalteu reagent using gallic acid as a standard. Briefly, after incubation at room temperature for 30 min, the absorbance of the mixture of CE and Folin–Ciocalteu reagent was measured at 750 nm by spectrophotometer. Total phenolic content of CE was expressed as mg of gallic acid equivalents (GAE) per gram of dried extract [16].

### In vitro antioxidant activity

#### DPPH radical scavenging activity

To perform DPPH assay, one milliliter of various concentrations of CE (0–25 μg/ml) was mixed with 2 ml of 0.1 mM DPPH in methanol and left standing for 1 h at room temperature in the dark. The absorbance was measured at 515 nm. Percentage of inhibition was calculated using the equation below. The antioxidant activity was expressed by the concentration required for 50% of scavenging of free radical (IC_50_) [17].

$$ \%\mathrm{inhibition}=\left[\left({\mathrm{Absorbance}}_{\mathrm{control}}-{\mathrm{Absorbance}}_{\mathrm{sample}}\right)/{\mathrm{Absorbance}}_{\mathrm{control}}\right]\times 100 $$

#### ABTS radical scavenging activity

The scavenging activity of CE against ABTS^•^ was measured as previously described with minor modifications [16]. ABTS radical cation (ABTS^•^) was produced by adding 14 mM ABTS solution to 4.9 mM potassium persulfate solution (1:1; *v*/v) for 16 h in the dark at room temperature. The 150 μl of CE at various concentrations (0–25 μg/ml), ascorbic acid or butylated hydroxytoluene (BHT) was added to 2850 μl of diluted ABTS^•^ solution, mixed and then incubated in the dark for 6 min. Finally, the absorbance of the reaction mixture was measured at 734 nm. The radical scavenging activity of CE was expressed by IC_50_ value.

#### Ferric reducing antioxidant power (FRAP) assay

FRAP assay was performed to investigate the reducing power of CE [18]. The absorbance of the resulting mixture was measured at 593 nm. The standard calibration curve was created using FeSO_4_·7H_2_O. The FRAP capacity of the extract was expressed as mM Fe^2+^ per gram extract.

### Experimental animals

Male Jcl:ICR mice of 6-week old (28–34 g), obtained from Nomura Siam International Co., Ltd., Bangkok, Thailand, were used in this study. Mice were housed in stainless steel cages lined with wood shavings at Laboratory Animal Facility, Suranaree University of Technology, under standard condition of 25 ± 2 °C, 45–50% relative humidity and 12-h light/dark cycle. Normal food and water were given ad libitum. The experiments were performed after 7 days of acclimatization. The extract was dissolved in distilled water and was administered to animal using oral gavage method. All mice were sacrificed by CO_2_ inhalation at the end of experiment and for tissue collection. All procedures were approved and conducted following the guidelines of the Institutional Animal Care and Use Committee, Suranaree University of Technology, Thailand (Approval number No. 1/2561).

### Oral glucose tolerance test in normal mice

The effect of CE on blood glucose level was first evaluated via oral glucose challenge. Six-hour fasted normal mice were randomly divided into 3 different groups (*n* = 6) as the following.

Group 1: normal mice + deionized water

Group 2: normal mice + 500 mg/kg CE

Group 3: normal mice + 10 mg/kg glibenclamide (Daonil®, Jakarta, Indonesia)

All mice were pretreated with drugs prior to the oral glucose administration at the dose 2 g/kg (2 h for CE and deionized water; 30 min for glibenclamide based on the onset of action from our preliminary study). Blood glucose level was monitored at 0, 30, 60 and 120 min from small incision of tail tip using Medisafe® EX glucose meter. The area under the curve was calculated using the same formula in the previous report [19].

### Hypoglycemic test in alloxan-induced diabetic mice

Overnight fasted mice were received an intraperitoneal injection of 130 mg/kg of alloxan monohydrate dissolved in cold 0.85% saline solution to induce type 2 diabetes [20]. Diabetic induction was checked after 3 days of alloxan injection. Mice showed glucose level greater than 200 mg/dl (survived without insulin) was considered as type 2 diabetic mice [21] and used for the experiment. Mice were randomly divided into 4 groups as follow.

Group 1: normal mice + deionized water (*n* = 6)

Group 2: diabetic mice + deionized water (*n* = 6)

Group 3: diabetic mice + 500 mg/kg CE (*n* = 6)

Group 4: diabetic mice + 10 mg/kg glibenclamide (*n* = 5)

After the single dose of drug administration, blood glucose levels were determined at 0, 1, 2, 4 and 6 h to evaluate acute hypoglycemic effect of the extract as described in the previous study [22].

### Effect of the extract on glucose absorption

The inhibitory effect of CE on glucose absorption was investigated using isolated mouse jejunum. The jejunum was isolated from normal mouse and placed in oxygenated Kreb-Henseleit solution (composition in g/l; NaCl 6.92, KCl 0.35, MgSO_4_.7H_2_O 0.29, CaCl_2_ 0.28, KH_2_PO_4_ 0.16, NaHCO_3_ 2.1, and D-glucose 1.4), pH 7.4. The jejunum was cut into 6 cm long segments, tied edges, everted and filled with Kreb-Henseleit solution. The sacs were incubated in 10 ml of Kreb-Henseleit solution containing each of the following substances CE (25 and 50 μg/ml) or acarbose (1 mg/ml) for 1 h in the presence of carbogen at 37 °C. Glucose concentrations inside the sacs were determined using PGO enzyme. Ten μl of diluted buffer was interacted with 190 μl of PGO enzyme solution in a microtitter plate. The reaction mixture was incubated at 37 °C in the dark for 30 min. The intensity of the brown color was measured at 450 nm using spectrophotometer. The concentration of glucose was calculated using standard curve of D-glucose. The amount of glucose absorption was calculated using the following formula [23].

$$ \mathrm{Amount}\ \mathrm{of}\ \mathrm{glucose}\ \mathrm{absorbed}=\left(\mathrm{amount}\ \mathrm{of}\ \mathrm{glucose}\ \mathrm{after}-\mathrm{amount}\ \mathrm{of}\ \mathrm{glucose}\ \mathrm{before}\right)/\mathrm{g}\ \mathrm{of}\ \mathrm{jejunum} $$

### Effect of the extract on glucose uptake

Glucose uptake by mouse abdominal muscle was measured as previously described [24]. Briefly, after animals were sacrificed, abdominal muscles were removed and soaked in the Kreb’s-Ringer bicarbonate (KRB) buffer, pH 7.4 with continuously supply of carbogen for 10 min. The muscle was then incubated with KRB buffer containing 200 mg/dl of D-glucose, CE 25 or 50 μg/ml with or without insulin (100 mU/ml) for 30 min. Then, buffer was collected and analyzed for the remaining glucose using PGO enzyme as described in the previous section. The amount of glucose uptake was calculated by the formula below.

$$ \mathrm{Amount}\ \mathrm{of}\ \mathrm{glucose}\ \mathrm{uptake}=\left(\mathrm{amount}\ \mathrm{of}\ \mathrm{glucose}\ \mathrm{before}-\mathrm{amount}\ \mathrm{glucose}\ \mathrm{after}\right)/\mathrm{g}\ \mathrm{of}\ \mathrm{muscle} $$

### Examination of the effect on α-glucosidase activity

The α-glucosidase inhibitory activity was measured as described previously [16]. Briefly, a mixture of 10 μl of 0.25 U/ml α-glucosidase (Sigma–Aldrich, USA), 50 μl of 0.1 M potassium phosphate buffer (pH 6.8) and 20 μl of various concentrations of the extract or the α-glucosidase inhibitor acarbose (Fluka, USA) was incubated at 37 °C for 10 min. Then, 10 μl of 5 mM p-nitrophenyl-α-D-glucopyranoside (PNPG) was added and further incubated for 30 min. To terminate the reaction, 50 μl of 0.1 M Na_2_CO_3_ was added. The absorbance was measured at 405 nm optically by using a spectrophotometer. Results were expressed as the concentration where the activity of α-glucosidase is inhibited by 50% (IC_50_).

### Statistical analysis

Each experiment was repeated at least 3 times and the result values were expressed as mean ± SEM.The comparisons between means were done using One way- or Two way-ANOVA followed by Student-Newman-Keuls. A value of *P* < 0.05 was considered as statistically significant differences.

## Results

### Phytochemistry and total phenolic content

The results of extract yield and phytochemicals screening were presented in Table 1. In this study, extract yield of *C. cainito* stem bark prepared by maceration method using water was 11.22 ± 0.54%. The phytochemical screening revealed the presence of phenols, tannin, glycosides, terpenoids, and saponin but the absence of flavonoids, alkaloids, and steroids. Total phenolic compounds found in the extract was 871.75 ± 10.41 mg GAE/g extract (Table 2).

**Table 1:** Phytochemical screening and yield of the aqueous extract of *C. cainito* stem bark

Test for	Results
Phytochemistry	
Phenols	+
Tannins	+
Glycosides	+
Terpenoids	+
Saponin	+
Flavonoids	–
Steroids	–
Alkaloids	–
Yield (%)	11.22 ± 0.54^a^

+ present; − absent

^a^Value is expressed as mean ± SEM (*n* = 3)

**Table 2:** Total phenolic content and antioxidant activities of *C. cainito* stem bark extract

	DPPH IC_50_ (μg/ml)	ABTS IC_50_ (μg/ml)	FRAP (mM Fe^2+^ /g extract)	Phenolic Content (mg GAE/g CE)
CE	4.66 ± 0.14^*^	2.10 ± 0.06^#^	291.56 ± 3.25	871.75 ± 10.41
AA	3.49 ± 0.12	1.86 ± 0.03	–	–
BHT	4.68 ± 0.03	5.07 ± 0.19	–	–

The values are expressed as mean ± SEM, *n* = 3. CE, AA, BHT were abbreviations of *C. cainito* extract, ascorbic acid and butylated hydroxytoluene, respectively

^*^P < 0.05 compared with AA; ^#^*P* < 0.05 compared with BHT by one-way ANOVA followed by Student-Newman-Keuls as post hoc test

### Antioxidant activity

**Fig. 1:**
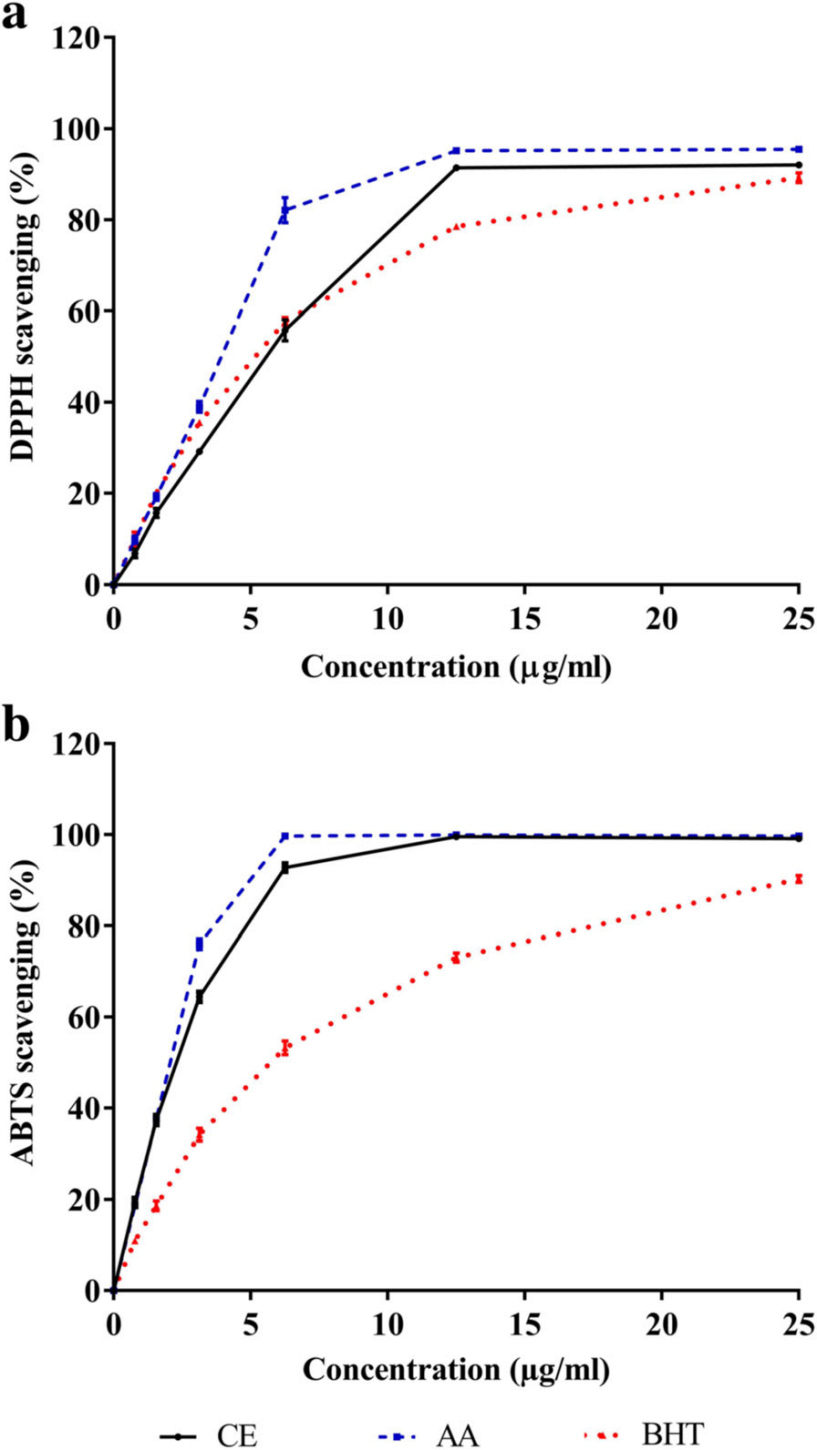
Antioxidant activity of *C. cainito* extract. Panel **a** and **b** are the results from DPPH and ABTS) radical scavenging methods, respectively. The values are expressed as mean ± SEM, *n* = 3. AA: ascorbic acid; BHT: butylated hydroxytoluene; CE: *C. cainito* extract

To access the antioxidant activity of the extract, DPPH, ABTS free radical scavenging and FRAP assay were performed. The concentrations of CE were varied from 0 to 25 μg/ml. The extract showed the maximum radical scavenging activity in the highest experimental concentration by 92% in DPPH assay and 99% in ABTS assay (Fig. 1). The IC_50_ values found for CE, ascorbic acid, and BHT from DPPH and ABTS assays were presented in Table 2. The reducing potential of CE was determined using FeSO_4_ standard curve. The FRAP value of CE was 291.56 ± 3.25 mM Fe^2+^ equivalent per gram of dried extract (Table 2).

### Oral glucose tolerance test in normal mice

**Fig. 2:**
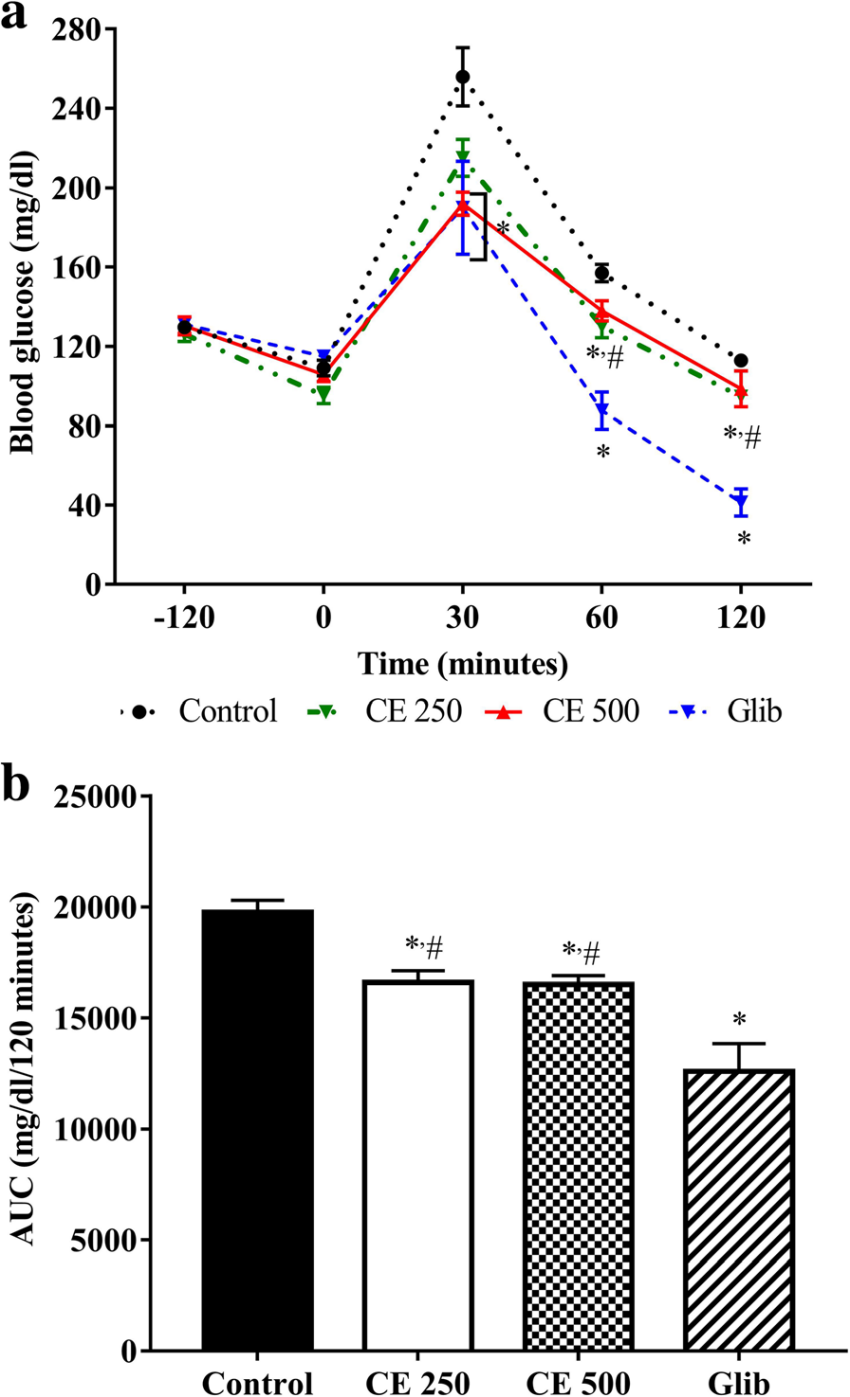
Effect of *C. cainito* extract on OGTT in normal mice. Panel **a** is blood glucose level during oral glucose challenge whereas Panel **b** is the area under the curve (AUC) of the blood glucose level over time. ^*^P < 0.05 compared with control mice; ^#^*P* < 0.05 compared with glibenclamide treated mice by one-way ANOVA followed by Student-Newman-Keuls as post hoc test. The values are expressed as mean ± SEM, *n* = 6. Glib: glibenclamide; CE: *C. cainito* extract

The results of the oral glucose tolerance test in normal mice are shown in Fig. 2. As seen in Fig. 2a, the initial blood glucose levels of all groups prior to drug administration were no difference. The blood glucose levels after glucose loading reached a peak at 30 min and decreased subsequently over time, in all groups. It was found that CE or glibenclamide had significantly improved glucose tolerance in normal mice. Mice received CE (500 mg/kg) and glibenclamide (10 mg/kg) noticeably suppressed the elevation of glucose after 30 min of glucose load compared to control group (*P* < 0.05). In addition, the area under the curve (AUC) was significantly reduced in both treated groups when compared to control mice (Fig. 2b).

### Antidiabetic effect of *C. cainito* extract in alloxan-diabetic mice

**Fig. 3:**
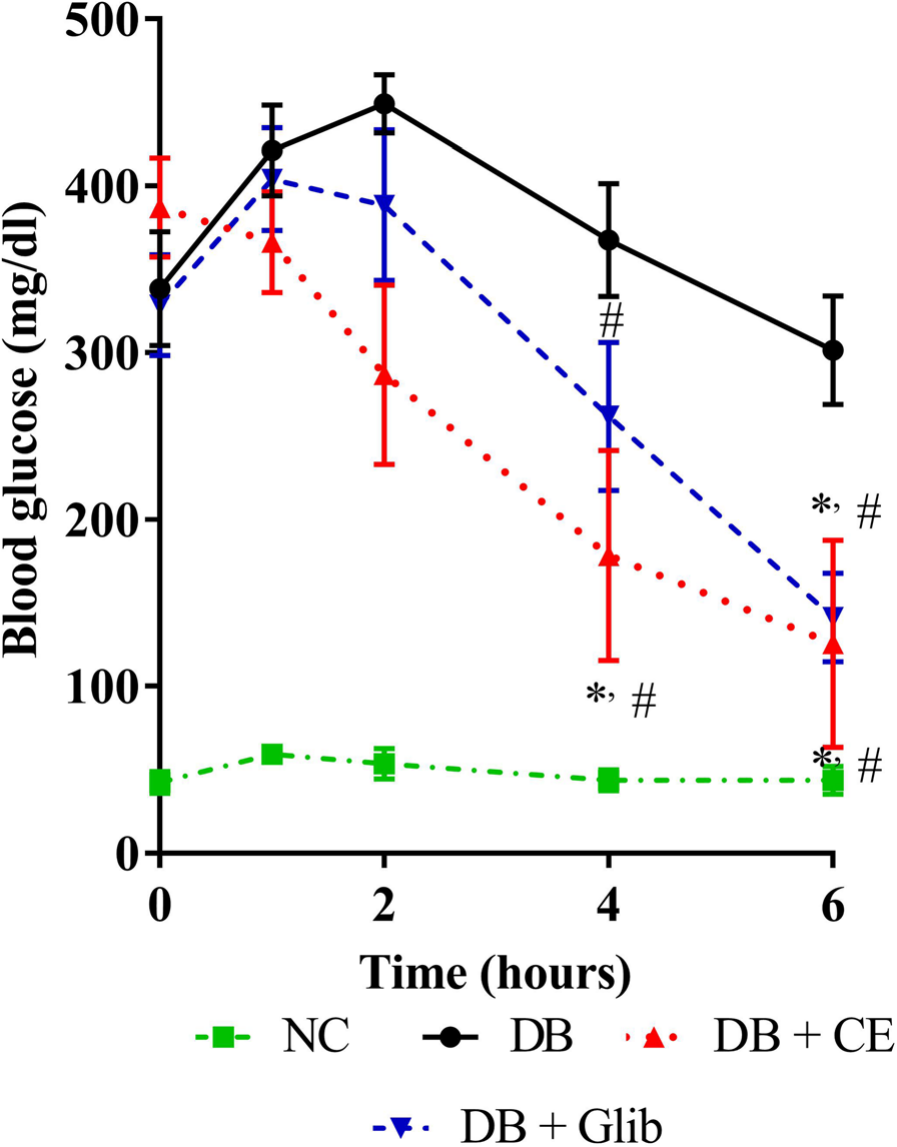
Acute effect of *C. cainito* extract on alloxan-induced diabetic mice. NC: normal control; DB: diabetic control; DB + CE: diabetes + CE 500 mg/kg; DB + Glib: diabetes + glibenclamide 10 mg/kg. ^*^P < 0.05 compared with diabetic control mice at the same time of experiment, ^#^*P* < 0.05 compared to the initial level in the same treatment by one-way ANOVA followed by Student-Newman-Keuls as post hoc test. The values are expressed as mean ± SEM, *n* = 5–6

Fig. 3 shows kinetics of blood glucose observed during the period of experiment. In this study, alloxan injection destroyed pancreatic β cells and reduced insulin secretion leading to an elevated blood glucose level compared to normal control mice. The extract and glibenclamide started to suppress the rise of blood glucose in diabetic mice after 2 h of treatment, but not statistically significant. However, after 4 h and longer blood glucose levels of the CE and glibenclamide groups declined significantly more than the diabetic control group (*P* < 0.05).

### Effect of *C. cainito* extract on glucose absorption

**Fig. 4:**
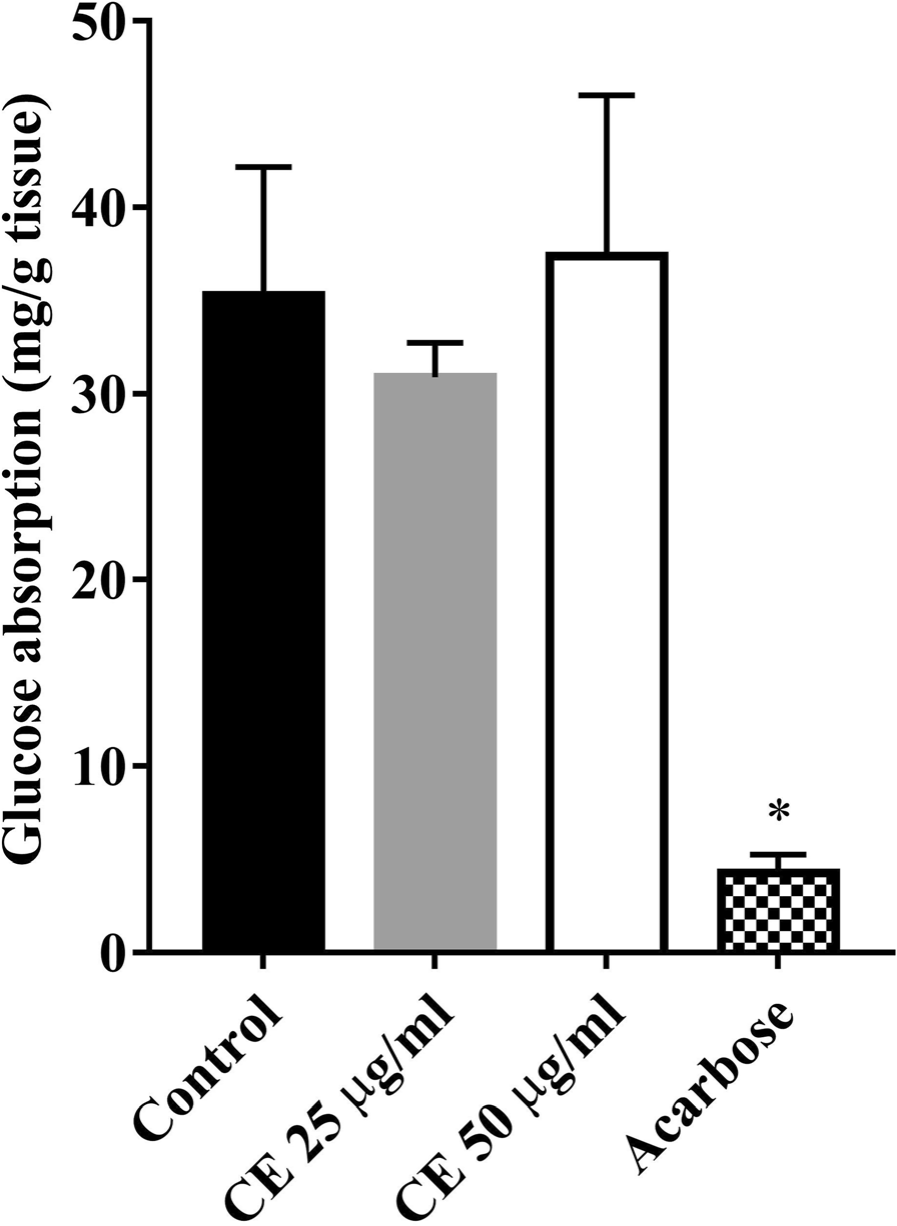
The effect of *C. cainito* extract (CE) on glucose absorption by everted mouse jejunum. The values are expressed as mean ± SEM, *n* = 5. ^*^P < 0.05 compared to control by one-way ANOVA followed by Student-Newman-Keuls as post hoc test

The everted sacs of the small intestines from mice were used for investigating the inhibitory effect of CE on glucose absorption ex vivo. The results shown in Fig. 4 indicated that the extract at the experimental concentrations (25 and 50 μg/ml) did not inhibit glucose absorption when compared to control. In contrast, acarbose at 1 mg/ml profoundly suppressed glucose absorption by everted sacs (*P* < 0.05).

### Effect of *C. cainito* extract on glucose uptake

**Fig. 5:**
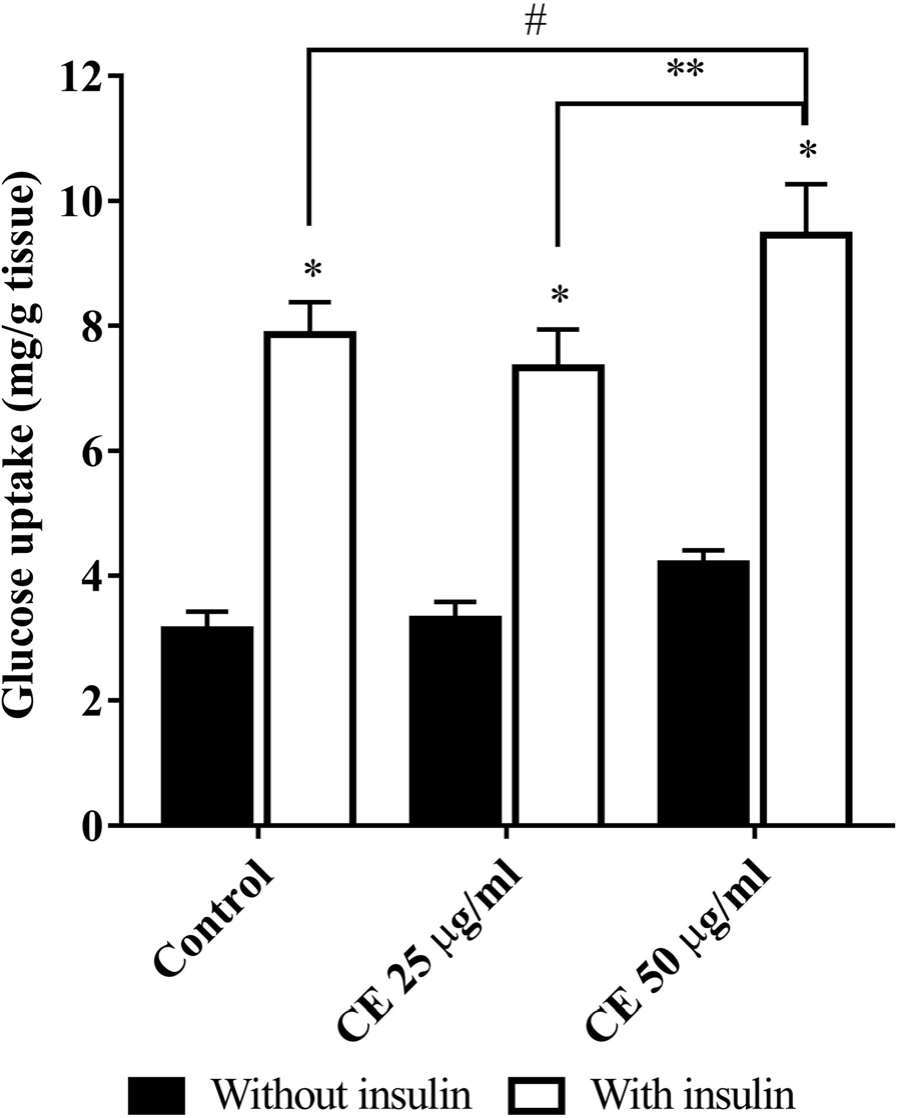
The effect of *C. cainito* extract (CE) on glucose uptake by isolated mice abdominal muscle. The values are expressed as mean ± SEM, *n* = 5. ^*^P < 0.05 compared with non-insulin in the same treated group, ^#^*P* < 0.05 compared with control, ^**^P < 0.05 compared with CE 25 μg/ml by two-way ANOVA followed by Student-Newman-Keuls as post hoc test

The effect of CE on glucose uptake is presented in Fig. 5. In normal group, low glucose uptake was found in the absence of insulin. Addition of insulin to the KRB buffer increased the glucose uptake significantly (*P* < 0.05). This effect was also observed in all experiments when compared to the non-insulin treated groups. The results showed that treatment of CE at 50 μg/ml with insulin significantly increased glucose uptake from 7.86 ± 0.52 (control) to 9.45 ± 0.82 mg/g tissue. However, without insulin, CE at the doses used had no significant effect on glucose uptake.

### α-Glucosidase inhibitory effect of *C. cainito* extract

**Fig. 6:**
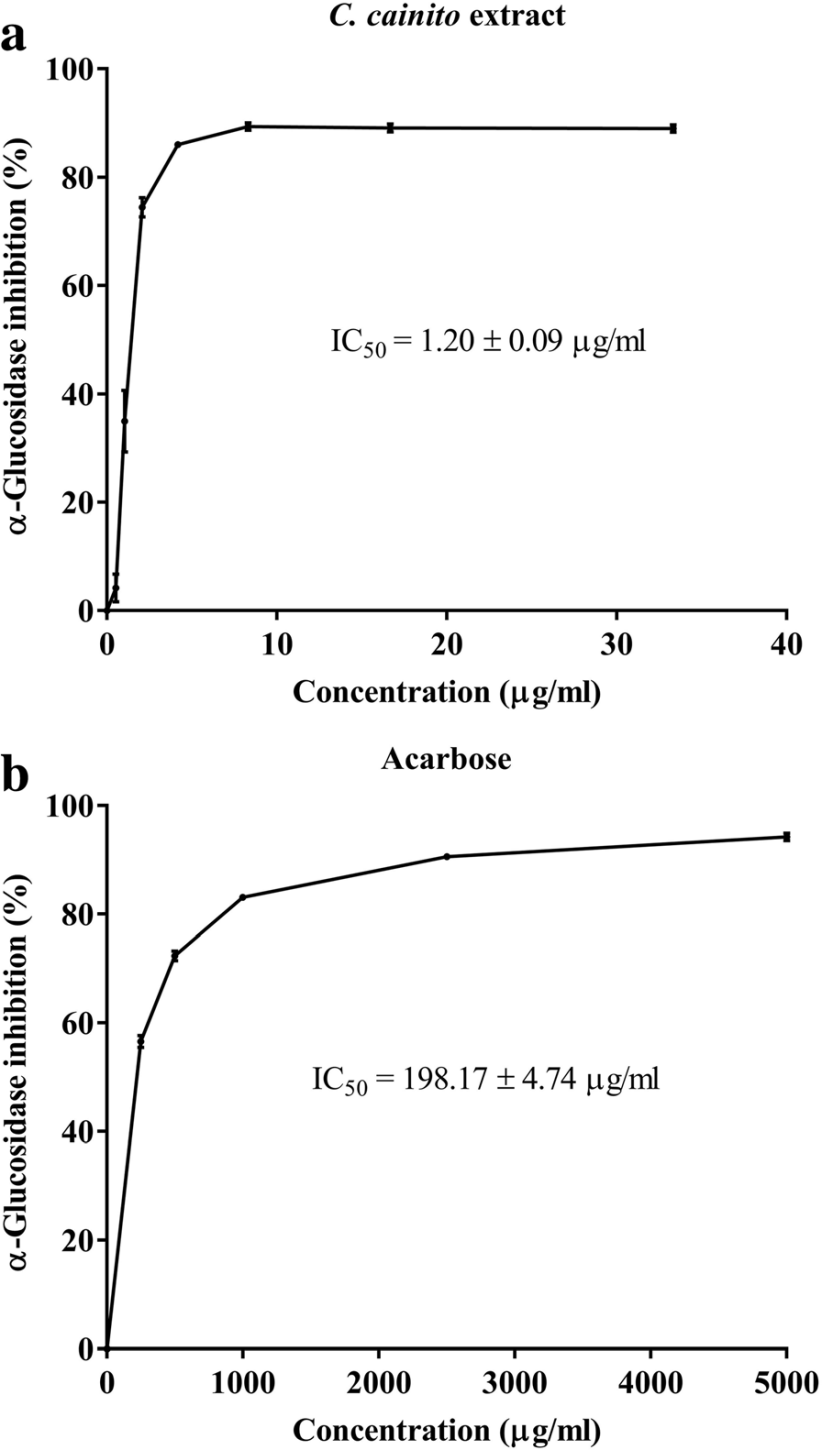
The effect of *C. cainito* extract (CE) on α-glucosidase activity. Values are expressed as mean ± S.E.M. of three separate experiments. Acarbose was used as positive control. Panel **a** and **b** are results from α-glucosidase inhibitory activities of *C. cainito* and acarbose, respectively. The calculated IC_50_ of the extract was 1.20 ± 0.09 μg/ml, whereas that of acarbose was 198.17 ± 4.74 μg/ml

α-Glucosidase enzyme is one of the medication targets in diabetic management. The enzyme is involved in digestion of polysaccharide into monosaccharide that can be absorbed by the intestine. In this study, α-glucosidase isolated from *Saccharomyces cerevisiae* was chosen as the target enzyme. The aqueous extract from *C. cainito* exhibited much greater inhibition on α-glucosidase activity compared to acarbose. The IC_50_ of CE was 1.20 ± 0.09 μg/ml whereas that of acarbose was 198.17 ± 4.74 μg/ml (Fig. 6).

## Discussion

It is widely accepted that the rapidly increasing incidence of diabetes mellitus has become a major health problem worldwide. The modern oral hypoglycemic agents such as sulphonylureas, biguanides, thiozolidinediones and α-glucosidase inhibitors are commonly used for the treatment of type 2 diabetes. However, it is well known that they can produce side effects associated with their applications [25]. Moreover, a progressive decline in their effectiveness, termed secondary failure have been reported [26]. During the past decade, there is a growing interest in alternative herbal medicine due to their efficacy, less side effects in clinical practice and relatively low costs. It has been estimated that about 800 plants have antidiabetic potentials [27]. Most of them have been used as folk medicines in many countries around the world. *C. cainito*, commonly called Star Apple in English and Vú Sữa (literally: milky breast) in Vietnamese, is one of medicinal plants which has long been prescribed by local practitioners for traditional treatment of diabetes mellitus. However, there is a paucity of scientific evidence that confirms its antidiabetic activity. Herein, we first evaluated the antidiabetic effect of the extract from *C. cainito* stem bark to confirm its benefits according to the use of this plant in Vietnam.

In this study, antidiabetic effect of the *C. cainito* extract was conducted in healthy and alloxan-diabetic mice. In normal mice, the hypoglycemic effect of the extract was investigated through an oral glucose tolerance test (OGTT). A 6-h fasting is considered as a best fasting duration for establishing an OGTT in mice [28]. The rise in blood glucose after 30 min confirmed successful oral glucose loading in every group (Fig. 2). The antidiabetic drug glibenclamide used in this study as a positive control reduces the postprandial hyperglycemia by increasing insulin secretion from β cell. Based on our preliminary experiment, the dose at 500 mg/kg was chosen for oral administration. As shown in Fig. 2a and b, the extract and glibenclamide improved glucose tolerance compared to vehicle control.

Alloxan, a toxic glucose analogue, enters pancreatic β-cell via GLUT2 glucose transporter. This chemical plays an important role in hyperglycemic animal model through its specific inhibition of glucokinase and stimulation of reactive oxygen species production, consequently causing necrosis and destruction of β-cells [29]. The data from Fig. 3 clearly showed that the administration of CE reduced blood glucose level in alloxan-induced diabetic mice similar to that of glibenclamide. These in vivo experiments provide the first scientific evidence supporting an anti-hyperglycemic activity of *C. cainito* stem bark.

Numerous mechanisms of action have been proposed for medicinal plants used in the treatment of diabetes mellitus. However, none has been postulated for antidiabetic activity of *C. cainito*. In this study, two possible mechanisms underlying its acute antidiabetic activity, an inhibition of glucose absorption and a stimulation of glucose uptake were examined. The glucose absorption was performed using everted jejunal sacs of mice. Acarbose, a well-known α-glucosidase inhibitor currently used for the treatment of diabetic patients, was used as a positive control because it has been shown to additionally inhibit the absorption of D-glucose from the intestinal lumen into the blood stream [23, 30]. The data in Fig. 4 showed that unlike acarbose, CE did not inhibit the glucose absorption.

Isolated skeletal muscle such as epitrochlearis muscle [31, 32] and abdominal muscle [24, 33, 34] have been exploited in the glucose uptake study. In the current study, we isolated the abdominal muscle from mice and incubated in a bicarbonate buffer with carbogen supplied constantly. The effect of CE on glucose uptake was evaluated in the absence and presence of insulin. The data in Fig. 5 shows that in the presence of insulin CE at 50 μg/ml significantly enhanced glucose uptake by the muscle (9.45 ± 0.82 mg/g tissue) compared to control (7.86 ± 0.52 mg/g tissue). Without insulin, CE treatment also showed an increase of glucose uptake, but not statistical significant. These in vitro experiments revealed that the extract promoted glucose transport in the skeletal muscle, especially in the presence of insulin, but no effect on glucose absorption. It is likely that the enhancement on glucose uptake contributes to the antidiabetic effect of CE.

In this study, the in vivo experiments were done only in the acute treatment mainly to provide scientific evidence to support the hypoglycemic activity of the extract. One may argue that the postulated action on glucose uptake in this study could not explain the acute anti-hyperglycemic effect of the extract. Skeletal muscle is recognized as the major site of insulin-mediated glucose uptake after carbohydrate consumption in human [35]. Insulin resistance is a hallmark of non-insulin dependent diabetic mellitus. Peroxisome proliferator-activated receptor gamma (PPARγ) is a key factor in insulin sensitivity. The activation of PPARγ by insulin sensitizers (e.g. thiazolidinediones) markedly improve the sensitivity to insulin, however, it requires long-term effect to cause gene expression change [36]. Moreover, PPARγ is prominently present in adipose tissue and nearly absent in muscle [37]. The enhancement of insulin action in skeletal muscle by CE found in this study tend to be mediated via other mechanism(s).

It is well established that glucose uptake by skeletal muscle is mostly via glucose transporter 4 (GLUT4). It has been shown that GLUT4 recruitment from cytosol to the cell surfaces of muscle can be acutely stimulated by both insulin and exercise independent of transcription or translation [38, 39]. The translocation of GLUT4 from intracellular vesicles to accumulate in the plasma membrane in the response to insulin was demonstrated to depend on the activation of the insulin receptor substrate 1, PI3K, PDK1 and Akt2 [40]. In addition, the data from a previous study suggested that an acute stimulation effect on insulin-mediated glucose uptake in skeletal muscle was related to the elevation of the phosphorylation and activation of key proteins involving in the translocation of GLUT4 such as Rac1, AS160 and Akt [41]. Other studies suggested that the rising in glucose uptake was due to the increase of AMPK phosphorylation [31, 42]. This pathway supports the acute anti-hyperglycemic effect of the CE by increasing glucose uptake in the muscle.

α-Glucosidase is a digestive enzyme which catalyzes the breakdown of polysaccharides into monosaccharide, the form of carbohydrate that the intestine can only be able to transport into the blood circulation. Therefore, the inhibition of α-glucosidase is one of the important approaches in oral antidiabetic medication. Reducing postprandial glucose level by delay glucose absorption after meal is the prominent benefit from α-glucosidase inhibitor [43]. In addition to the glucose uptake stimulation which is proposed as a mechanism underlying the acute hypoglycemic effect of *C. cainito* extract in this study, α-glucosidase inhibitory effect was also investigated. The IC_50_ value of the extract was found at 1.20 ± 0.09 μg/ml, approximately 200 times lower than that of acarbose which is used clinically as antidiabetic drug. Although this action could not be attributed to the anti-hyperglycemic mechanism in this study, it could be anticipated to contribute to the blood lowering effect when used as alternative medicine in diabetic patients. Moreover, this plant could be a potential candidate for a search of a new α-glucosidase inhibitory drug for diabetic care.

Free radicals are found to be associated with many diseases, including diabetes. In diabetes mellitus, the supplement of antioxidant agents show promising effect on the reverse of the oxidative stress biomarkers and diabetic complications [3]. Major antioxidant ability can be classified into two groups, hydrogen atom transfer and single electron transfer based assays. For in vitro antioxidant measurement, a single assay may not provide sufficient evidence for antioxidant potential of a compound. Therefore, in this study, the antioxidant activity of CE was assessed by different methods including DPPH, ABTS, and FRAP assays. DPPH, a stable organic nitrogen radical, is used to perform a simple technique assay. DPPH assay has been considered as a valid accurate and easy method to determine radical scavenging activity of antioxidants. However, the disadvantages of this method are (1) the cross interaction between DPPH radical and other radicals and (2) the time response curve to reach the final stable state is not linear with different ratios of antioxidant and DPPH [44]. In ABTS assay, the oxidant was generated by the reaction between ABTS ammonium and potassium persulfate. This assay has been used commonly in many laboratories although it costs time to prepare the radical. The FRAP assay is an electron transfer based assay. The oxidant involves many Fe (III) species and many metal chelators in food extract. The redox potential of Fe (III) salt in FRAP assay is comparable to ABTS^•^ in ABTS assay [45]. As shown in Table 2, the results from all methods used in this study were in agreement that the extract had quite strong antioxidant activity.

Phytochemical analysis of CE obtained from this study showed the presence of phenols, tannin, glycosides, terpenoids, and saponin of which the antidiabetic effects have been established [46–49]. The extract contains great amounts of phenolic compounds (871.75 ± 10.41 mg GAE/g extract) and had high antioxidant potential comparable to antioxidant power of the standard antioxidant ascorbic acid and butylated hydroxytoluene (Table 2). These results agree with previous report that high phenolic content was correlated with strong antioxidant activity [48]. It can be anticipated that the antioxidant activity of CE may be beneficial in the long-term treatment of diabetic patients. Nine polyphenolic compounds, (+)-catechin, (+)-gallocatechin, (−)-epigallocatechin, quercetin, quercitrin, isoquercitrin, myricitrin, gallic acid and (−)-epicatechin were isolated from fruit of *C. cainito* [49]. Others including ursolic acid, β-sitosterol, lupeol and gallic acid were extracted from the leaves [50]. It has been demonstrated that these compounds possess antidiabetic activity [51–54]. In terms of the compositions of *C. cainito* stem bark, there is lack of information. Phytochemical verification and identification of the active ingredients responsible for its antidiabetic activity need further investigation.

## Conclusion

In conclusion, the aqueous extract from *C. cainito* stem bark possesses a strong in vitro antioxidant activity and in vivo antidiabetic effects. It is postulated that the mechanism of action contributing the acute anti-hyperglycemic effect of the extract is the enhancement of glucose uptake by the muscles. Moreover, the extract was also found to possess strong α-glucosidase inhibitory effect which may contribute to its anti-hyperglycemic action when used in diabetic patients. The results obtained in the present study provide scientific rationale to corroborate the use of *C. cainito* stem bark for its traditional diabetic treatment.

## Abbreviations

AA: Ascorbic acid
ABTS: 2,2′-azino-bis (3-ethylbenzothiazoline-6-sulfonic acid) diammonium salt
BHT: Butylated hydroxytoluene
CE: *C. cainito* extract
DPPH: 2,2-diphenyl-1-picrylhydrazyl
FRAP: Ferric reducing antioxidant power
GAE: Gallic acid equivalent
KRB: Kreb’s-Ringer bicarbonate
OGTT: Oral glucose tolerance test
PGO: Peroxidase-glucose oxidase
